# Perceptions, challenges and determinants of management strategy choices for combating fall armyworm ( *Spodoptera frugiperda* ) infestation in Ghana

**DOI:** 10.1002/ps.70956

**Published:** 2026-05-17

**Authors:** Richard Adabah, Robert Aidoo, Bright Owusu Asante, Amos Mensah, Seth Etuah, Gifty Boakye Appiah, Jonas Osei‐Adu

**Affiliations:** ^1^ Department of Economics Swedish University of Agricultural Sciences Uppsala Sweden; ^2^ Council for Scientific and Industrial Research – Crops Research Institute Kumasi Ghana; ^3^ Department of Agricultural Economics, Agribusiness and Extension Kwame Nkrumah University of Science and Technology, KNUST Kumasi Ghana

**Keywords:** determinants, fall armyworm, multivariate probit, perception, pest control measure

## Abstract

**BACKGROUND:**

This study assessed the perceptions and management strategy choices of maize farmers in Ghana towards fall armyworm (FAW) infestation, which poses a severe threat to their livelihoods, and examined the factors influencing farmers' adoption of FAW management strategies.

**RESULTS:**

All respondents demonstrated high awareness of FAW, with 98% reporting devastating effects on their farms. FAW affected an average of 30% of maize farmland, with farmers losing an estimated 536 kg ha^−1^, representing approximately 27% of expected maize yield. A significant knowledge gap persists in effective control measures, as indicated by a mean perception index of 0.21, reflecting farmers' limited understanding of effective FAW management strategies. Relative to biological/botanical and traditional pest management strategies, farmers largely perceive synthetic pesticides to be the ideal solution, with 42% strongly agreeing and 25% agreeing with this view. Factors significantly influencing control measure choice included age, gender, household size, labour, household head status, farm size, farm experience and non‐farm income. The most pressing challenge with FAW management was difficulty in accessing synthetic control measures.

**CONCLUSION:**

Pest control practices vary significantly across agroecological zones, suggesting the need for region‐specific strategies. Despite high awareness of FAW, farmers lacked adequate knowledge of effective control strategies, with many applying pesticides based on hearsay rather than expert recommendations. These findings underscore the need for targeted training programs focused on improving farmers' understanding and adoption of integrated, sustainable FAW management practices, while addressing the misguided perception that synthetic pesticides are inherently superior. © 2026 The Author(s). *Pest Management Science* published by John Wiley & Sons Ltd on behalf of Society of Chemical Industry.

## Graphical abstract

Despite high awareness of fall armyworm, a significant gap remains in the use of sustainable and effective control measures. Farmers predominantly perceive synthetic pesticides as ideal despite studies highlighting botanical and traditional methods as more sustainable. Adoption is shaped by education, farm and household characteristics, while accessibility, knowledge gaps and cost remain key barriers.

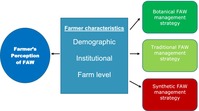

## 1 INTRODUCTION

Fall armyworm (FAW), *Spodoptera frugiperda* (Lepidoptera: Noctuidae), is a severe pest that damages a range of crops, including maize, rice, sorghum and vegetables.^1^ It attacks all stages of maize, from seedlings to mature plants, causing significant reductions in both yield quantity and quality.^2^ Originally from tropical and sub‐tropical America, FAW was first reported in sub‐Saharan Africa in late 2016, with Ghana, Nigeria, Togo and Benin among the earliest affected countries.^3^ By 2020, FAW had spread across all African countries except Djibouti, presenting a serious threat to maize production and food security across the continent.^4^

Understanding farmers' perceptions and decision‐making processes is crucial in managing pest infestations effectively. Factors such as attitudes, perceived behavioural controls and norms significantly influence how farmers respond to pests and choose management strategies.^5^ In Ghana, maize farmers use a variety of control methods for FAW which are categorised into three main groups: synthetic, biological/botanical and traditional/cultural control measures. Synthetic control measures comprise the use of commercially formulated chemical pesticides. Biological/botanical measures encompass the use of natural enemies of FAW (particularly parasitoids) and plant‐based extracts, with neem‐based preparations being the most commonly mentioned by farmers. Traditional/cultural measures consist of agronomic practices aimed at controlling FAW presence and damage, such as crop rotation, intercropping maize with legumes, fallow periods, push‐pull technology, timely weeding, hand‐picking of larvae, slashing and burning of infested plants, among others. The adoption of these methods is closely linked to farmers' perceptions and the underlying factors driving their choices.^6^, ^7^

Existing research highlights a range of FAW control measures. Advanced pest control techniques, such as genetically modified organisms and biological controls, are less accessible to African farmers, who primarily rely on conventional methods.^8^, ^9^ In Brazil, farmers use biological and botanical methods, including genetically modified organisms and natural predators.^8^ In contrast, African farmers often rely on chemicals, botanical pesticides and cultural practices due to the limited availability of advanced methods.^10^, ^11^, ^12^ Studies show varying perceptions of these methods' effectiveness, with some farmers finding synthetic pesticides inadequate,^10^ while others view them as essential.^12^

Despite the use of these methods, there is a significant gap in empirical research regarding farmers' perceptions of FAW, the factors influencing their choice of control strategies and the challenges they encounter. This study addresses this gap by providing empirical data on how farmers perceive FAW and the determinants of their control measures. The study therefore predicts farmers' perceptions of fall armyworm and their choice of control measures are significantly influenced by certain socioeconomic, farm and institutional factors. Additionally, limited access to resources and knowledge gaps are predicted to hinder the adoption of effective control practices.

Study results give a comprehensive analysis of the factors driving the adoption of different FAW control methods among maize farmers in Ghana. By identifying and examining these key factors, the study offers new insights into pest management strategies in developing countries. It contributes to the fight against pest invasions by revealing the importance of socioeconomic and perceptual factors in pest management and providing actionable recommendations for enhancing FAW control practices. Further, findings aim to guide policymakers and stakeholders in developing effective strategies to improve adoption of sustainable practices and agricultural productivity in regions affected by FAW.

## 2 MATERIALS AND METHODS

### 2.1 Study area

This study was carried out in four agroecological zones across four regions in Ghana. The study areas were the transitional agroecological zone in the Bono East region, the semi‐deciduous forest zone in the Ashanti region, the Guinea savannah in the Northern region, and the Sudan savannah in the Upper West region. These regions were selected due to their significant contributions to Ghana's maize production.

The transitional agroecological region of Ghana features a wet semi‐equatorial climate and woodland vegetation. The semi‐deciduous agroecological zone has fertile soils, creating an ideal environment for cultivating various crops. The Guinea savannah zone is arid, with predominantly grassland vegetation and drought‐tolerant trees. The Sudan savannah is characterised by short, drought‐tolerant and fire‐resistant deciduous trees, along with scattered grasslands. Unlike the bimodal rainfall pattern in the transitional and semi‐deciduous zones, the Guinea and Sudan savannah zones experience unimodal rainfall.

### 2.2 Sample selection and data collection method

The research used a mixed data collection approach (quantitative and qualitative) in collecting primary data. A standardised structured questionnaire was used for face‐to‐face interaction to collect quantitative data on the demographic, farm and institutional characteristics of respondents. Also, the focus group discussions and inputs from key informants helped in collecting data on farmers' perceptions and management practices.

The target population for this study was all maize farmers registered with a farmer‐based organisation, an innovation platform and with agricultural extension agents in the study areas. Following sample size estimation methods,^13^, ^14^ the minimum sample size was determined. The formula specification is as follows:

$$n=\frac{Z^{2}\times p\times q\times N}{\left[e^{2}\left(N-1\right)\right]+\left(Z^{2}\times p\times q\right)}$$

where *n* is the required sample size, *Z* ^2^ is the abscissa of the normal curve that cuts off an area *α* at the tails (1 − *α* equals the desired confidence level, the value for *Z* is found in statistical tables which contain the area under the normal curve, e.g. *Z* = 1.96 at the 95% confidence level), *N* is the population size of maize farmers in the study area, *p* is the population proportion of maize farmers with infested fields (assumed to be 0.5 since this would provide the maximum sample size), *q* is 1 – *p* and e is the desired level of precision or margin of error (5% error or 0.05).

The estimated minimum sample (*n*) from the formula was 294, but the actual sample size used was 474 respondents. The larger sample size allowed for smaller margins of error and improved statistical power, which increased the reliability and accuracy of the results. A multistage sampling technique was used. This involved using different sampling procedures at the various stages of the sampling process. At the first stage the four regions, Bono East, Ashanti, Northern and Upper West, were purposively selected. Then, two districts were purposively selected from each region. The above‐mentioned regions and respective districts were chosen purposively due to their significant contribution to maize production in the country.^15^, ^16^ Table 1 summarizes the sampling procedure showing the districts chosen and the number of respondents chosen from each district.

**Table 1:** Sampling procedure

Region	Agro‐ecology	Districts (sample)		Total
Bono East	Transition zone	Techiman (91)	Nkoransa (60)	151
Ashanti	Semi‐deciduous forest	Ejura (71)	Mampong (64)	135
Northern	Guinea savannah	Zabzugu (42)	Tatale‐Sanguli (51)	93
Upper West	Sudan savannah	Sissala East (64)	Sissala West (31)	95
				**Total = 474**

A total of 474 respondents were randomly drawn from four communities per district to form the sample for the study. The sampling strategy allowed for a representative sample of maize farmers in the selected districts.

### 2.3 Data analysis

Both descriptive and inferential statistical methods were utilised to analyse the data using STATA software. The data analysis was tailored to capture agroecological variations, highlighting differences in FAW infestations across distinct zones. As De Groote^17^ emphasises, FAW infestation varies with agroecology, necessitating the need for region‐specific solutions to effectively address the menace. This approach not only enhances localised interventions but also provides valuable insights for countries with similar agroecological conditions. Descriptive statistics, such as means, frequencies and percentages, were used to summarise the socio‐demographic, farm and institutional characteristics of the respondents. To test for significant differences among farmers across different agroecological zones, an analysis of variance was performed. This combination of statistical methods allowed for a comprehensive analysis of the data and facilitated the identification of important patterns and trends.

#### 2.3.1 Assessment of farmers' perception of FAW infestation

Understanding farmers' perceptions of pest infestation and management is essential for developing more effective, sustainable and environmentally responsible pest management strategies that benefit both farmers and the broader community. Farmers' perceptions of FAW management were assessed using perception index analysis based on Likert‐scale responses. Likert‐type scales are widely used to measure attitudes, opinions and perceptions by asking respondents to indicate their level of agreement or disagreement with a set of statements.^18^, ^19^, ^20^ This method allows for the quantification of individuals' perceptions, takes into account multiple dimensions, provides an objective approach of analysing views and produces easily interpretable results. The perception index is a composite measure that converts responses to perception statements, captured through a Likert scale, into numerical scores that quantify individuals' views on a subject.^21^, ^22^ In this study, a five‐point Likert scale was employed to rank farmers' responses to perception statements on FAW management to obtain the perception indices. The statements were designed to explore various dimensions of farmers' perceptions, such as awareness of the fall armyworm, perceived risk and efficacy of management practices. The Likert scale ranged from strongly agree (1) to strongly disagree (−1). The weighted mean for each statement was thus calculated as:

$$\text{weighted mean}=\frac{-1\times n_{\mathrm{sd}}+-0.5\times n_{d}+0\times n_{e}+0.5\times n_{a}+1\times n_{\mathrm{sa}}}{N}$$

where *n*_*i*_ is the number of respondents for each agreement level (strongly disagree to strongly agree), *N* is the total number of respondents, and sd, d, e, a, and sa indicate strongly disagree, disagree, neutral, agree and strongly agree, respectively.

The mean perception index was then estimated by the summation of weighted means (WM) divided by the number of perception statements (*N*_p_). The perception index is estimated as:

$$\text{perception index}=\frac{\Sigma \mathrm{WM}}{N_{p}}$$

where WM is the estimated weighted means and *N*_p_ is the number of perception statements.

The perception index ranges from −1 to 1, where values between −1 and −0.5 indicate a negative perception, a value of 0 represents a neutral perception, and values between 0.5 and 1 indicate a positive perception.

#### 2.3.2 Multivariate probit regression model

A simple tabulation multiple response analysis was used to analyse the various management strategies employed by maize farmers in controlling FAW infestation. Further, the factors influencing farmers' choice of FAW management strategies were analysed using the multivariate probit (MVP) regression model. The MVP is an appropriate method to estimate correlated binary outcomes simultaneously.^24^ This capability makes it an ideal choice for analysing situations where multiple outcomes can occur together and these outcomes may influence each other. In the context of pest management, this model allows for an in‐depth understanding of how farmers decide between different management practices, considering that these practices may not be mutually exclusive and could be adopted in combination. Multiple management practices are available to farmers in curbing FAW infestation. This study thus assumes that farmers adopt these multiple management techniques simultaneously and these techniques are not mutually exclusive. For instance, a farmer might use chemical pesticides in conjunction with crop rotation and biological controls to manage FAW infestation. The MVP was thus considered as it allows for possible concurrent correlation because farmers can choose to adopt different management practices simultaneously. This model has been used in simultaneity adoption by several authors.^25^, ^26^, ^27^ In line with previous authors, the MVP for management practice *i* was specified as

(1) $${Y_{i}}^{*}=\beta_{i}X_{i}+e_{i}$$

where ${Y_{i}}^{*}$ is the dependent variable (management practice) with *i* denoting a type of management practice (*i =* 1, synthetic; 2, biological/botanical; 3, traditional). It is assumed that a farmer adopts or uses a particular management practice (*Y* = 1) if $\;{Y_{i}}^{*}$ > 0, and does not use a management practice (*Y* = 0) if$\;{Y_{i}}^{*}$ ≤ 0. *X*_*i*_ is the factors affecting the choice of management practice *I*, *β*_*i*_ is the unknown parameters to be estimated and *e*_*i*_ is the random errors for management practice *i*.

The explanatory variables included in the MVP model, along with their measurement and *a priori* expectations based on empirical evidence, are summarised in Table 2.

**Table 2:** Variable definition and *a priori* signs

		Expected signs		
Explanatory variables	Measurement	Synthetic control	Biological/botanical control	Traditional control
Age	Years	+	+/−	+
Sex	Dummy (1 = male, 0 = female)	+	+/−	+
Household head status	Dummy (1 = yes, 0 = no)	+/−	+	+
Household size	Number of members	+	+	+
Marital status	Dummy (1 = married, 0 = unmarried)	+	+	+
Years of education	Years	−	+	+
Farming experience	Years	+	+	+
Maize farm size	Area planted in hectares	+/−	+	+/−
Land tenure	Dummy (1 = own land, 0 = other)	+	+	+
FBO/IP membership	Dummy (1 = yes, 0 = no)	+	+	+
Extension access	Dummy (1 = yes, 0 = no)	+	+	+
Attendance to pest and diseases training	Dummy (1 = yes, 0 = no)	+	+	+
Access to credit	Dummy (1 = yes, 0 = no)	+	+	−
Non‐farm income	Yearly income (Cedis)	+	+	−
Labour	Man‐days per hectare	+	+	+
Transition zone	Dummy (1 = farmer location; 0 = otherwise)	+/−	+/−	+/−
Semi‐deciduous forest zone	Dummy (1 = farmer location; 0 = otherwise)	+/−	+/−	+/−
Guinea savannah zone	Dummy (1 = farmer location; 0 = otherwise)	+/−	+/−	+/−

FBO, farmer‐based organization; IP, innovation platform.

#### 2.3.3 Kendall's ranking technique

Evaluation of the constraints associated with managing FAW among maize farmers was analysed using the Kendall's ranking technique. The evaluation of constraints associated with managing FAW among maize farmers utilising the Kendall's ranking technique, as referenced by Mattson,^28^ offers a systematic approach to understanding the challenges faced by farmers. This method provides insights into the relative importance of various constraints, as perceived by the farmers themselves. Kendall's *W* is computed as:

$$W=\frac{12\sum R_{j}^{2}-3m^{2}n{\left(n+1\right)}^{2}}{m^{2}\left(n^{3}-n\right)}$$

where W is Kendall's coefficient of concordance, m is the number of respondents, n is the number of constraints and $R_{j}$ is the sum of ranks assigned to constraint *j*.

Each constraint's mean rank was calculated as the average rank assigned to each constraint across all respondents. It is calculated by summing the ranks given by all respondents for a specific constraint and then dividing this sum by the total number of respondents, reflecting the overall consensus on its importance. A lower mean rank indicates that the constraint is more important or significant among farmers and *vice versa*. The coefficient of concordance (*W*), also known as the Kendall's *W*, is a non‐parametric statistic used to measure the agreement among rankings. It ranges from 0 (no agreement) to 1 (complete agreement). Subsequently, a higher Kendall's *W* denotes high level of agreement on the rankings. Thus, a high *W* value signifies strong consensus among farmers, indicating that their ranking of the constraints is similar. In contrast, a low *W* value reveals inconsistencies in the rankings, reflecting weak agreement among the farmers.

## 3 RESULTS AND DISCUSSIONS

### 3.1 Socio‐economic characteristics of farmers

Table 3 presents the summary statistics of sampled maize farmers by agroecological zones. In general, the average age of a maize farmer interviewed was 42 years, with significant differences across the agroecological zones. This depicts a mid‐age active maize farming population. Males significantly dominated maize farming, representing 70% of the total sample. A typical maize farming household consisted of eight members. Regarding formal education, the average years of schooling among respondents was 5 years, indicating a low literacy rate among maize farmers, a characteristic common to farmers in Ghana. The literacy rate varied significantly across agroecological zones at the 1% significance level. An average maize farmer cultivated on 2.6 ha of arable land with statistically significant variation across agroecological zones. In general, farmers had an average of 14 years of experience in maize cultivation, indicating a relatively high level of farming experience among respondents.

**Table 3:** Summary statistics of respondents across agroecological zones

Variable	Agroecological zones					*F*‐*Stat*
	Transition (*n* = 151)	Semi‐deciduous forest (*n* = 135)	Guinea savannah (*n* = 93)	Sudan savannah (*n* = 95)	Pooled (*N* = 474)	
	Mean (SD)	Mean (SD)	Mean (SD)	Mean (SD)	Mean (SD)	
Age	41.4 (11.75)	46.7 (12.86)	39.4 (14.39)	39.9 (13.83)	42.3 (13.33)	7.93***
Sex (male = 1)	0.84 (0.36)	0.67 (0.47)	0.58 (0.50)	0.61 (0.49)	0.70 (0.46)	8.53***
Household head (yes = 1)	0.79 (0.41)	0.76 (0.43)	0.55 (0.50)	0.40 (0.49)	0.66 (0.47)	18.31***
Household size	6 (5.77)	8 (5.56)	8 (5.06)	9 (5.89)	8 (5.68)	2.82**
Years of education	5.8 (4.80)	6.5 (5.26)	3.5 (5.21)	3.8 (5.27)	5.1 (5.14)	9.65***
Maize area cultivated (hectare)	3.1 (9.25)	2.6 (4.93)	2.5 (6.56)	2.2 (2.71)	2.6 (6.44)	8.29***
Farming experience	16 (11.26)	15 (11.42)	12 (11.44)	11 (10.82)	14 (11.39)	4.56***
FBO membership (yes = 1)	0.58 (0.49)	0.75 (0.44)	0.75 (0.43)	0.52 (0.50)	0.65 (0.48)	7.10***
Extension access (yes = 1)	0.53 (0.50)	0.52 (0.50)	0.71 (0.46)	0.47 (0.50)	0.55 (0.50)	4.34***
Distance to nearest extension agent (km)	19.6 (9.08)	10.4 (7.41)	5.2 (2.94)	8.2 (6.67)	11.4 (8.94)	74.16***
Distance to market (km)	13.3 (8.73)	12.8 (7.62)	5.8 (2.71)	7.8 (4.31)	10.5 (7.51)	83.99***
Participated in pest and diseases training (yes = 1)	0.74 (0.44)	0.84 (0.37)	0.66 (0.48)	0.51 (0.50)	0.70 (0.46)	9.76***
Off‐farm income (GH₵)	2317 (0.98)	2672 (1.17)	2119 (0.84)	3271 (0.91)	2577 (1.02)	0.92
Credit access (yes = 1)	0.13 (0.33)	0.10 (0.31)	0.38 (0.49)	0.48 (0.49)	0.22 (0.41)	16.83***

***1% significance level; **5% significance level; *10% significance level. FBO, farmer‐based organization; GHC, Ghana cedis; SD, standard deviation.

About 65% of the respondents were members of a farmer‐based organisation. Farmer groups are important sources of ideas as they tend to learn from each other, take decisions on farming methods and share information through social interactions. The average distance travelled by a farmer to gain main market access was estimated at 10.5 km with regards to the pooled sample. Whereas farmer access to extension services was quite appreciable (55%), a farmer had to travel an average of 11 km to access one. The estimated extension to farmer ratio in Ghana is 1:1850,^29^ indicating that a single extension officer within a planting season (3–4 months) is expected to visit nearly 1900 allocated farmers.

Further, farmer participation in insect pest‐ and disease‐focused training was encouraging, with 70% of respondents in attendance. This high attendance can lead to a conclusion that farmers are in dire need of pest and disease management measures to curb the insect pest and its devastating activities. These trainings are typically delivered as short group sessions (half‐day to 1‐day events) organised at community or farmer‐group level and facilitated by extension agents from the Ministry of Food and Agriculture, as well as by non‐governmental organizations. They cover topics on pests and diseases, ranging from identification of common pests and symptoms of infestation to appropriate control measures, safe pesticide handling and integrated pest management practices. Training participation was not consistent across the various agroecological zones, with significant differences observed at the 1% significance level. Moreover, differences between trained and untrained farmers have important implications for the interpretation of the estimated effect of FAW‐related training. Farmers who receive training are more likely to be better educated, to belong to farmer groups, to have contact with extension agents and to cultivate relatively larger farms.^23^ These patterns are consistent with positive selection into training and indicate that trained farmers may already be different from untrained farmers in ways that are related to FAW management outcomes. Although our regression models control for a wide range of farmer and farm‐level characteristics, unobserved factors, such as motivation, risk preferences or managerial ability, may still influence both participation in training and outcomes. As a result, the estimated coefficient should be interpreted as associations rather than strict causal effects. Farmers involved in off‐farm income activities generated an average of GH₵2577 annually, equivalent to US$170 (rate as at 2024). With regards to credit for farming activities, credit access was limited among maize farmers, with only 22% of respondents successfully securing credit to finance their farming activities.

### 3.2 FAW knowledge and infestation among farmers across agroecological zones

Table 4 presents the knowledge level and fall armyworm infestation among maize farmers across the agroecological zones. Pest and disease management is more effective when farmers' knowledge levels are taken into account, therefore it is important to understand farmers' knowledge of FAW and its infestation. FAW has now become a household name among Ghanaian farmers as all respondents (100%) were able to identify the crop pest. The average maize farmer had suffered six FAW infestations since its outbreak in Ghana in late 2016. The Guinea and Sudan savannah zones recorded fewer FAW infestations compared to the Transitional and Semi‐deciduous forest zones, likely due to their single growing season, unlike the bimodal rainfall pattern in the southern and middle belts of Ghana, which allows for two growing seasons. Table 4 also shows the proportion of maize farmland infested by FAW, with an overall mean infestation of 30.1%, which varied significantly across zones at the 1% level. This justifies reports by previous studies^17^ that prevalence of FAW is agroecologically informed and hence approaches to managing the pest should consider agroecology of farmers.

**Table 4:** FAW knowledge and infestation across agroecological zones

Variable	Agroecological zones					*F*‐*Stat*
	Transition (*n* = 151)	Semi‐deciduous forest (*n* = 135)	Guinea savannah (*n* = 93)	Sudan savannah (*n* = 95)	Pooled (*N* = 474)	
	Mean (SD)	Mean (SD)	Mean (SD)	Mean (SD)	Mean (SD)	
FAW identification (yes = 1)	1 (0)	1 (0)	1 (0)	1 (0)	1 (0)	‐
Number of FAW infestations (since first infestation)	6 (1.22)	8 (1.67)	4 (0.60)	4 (0.55)	6 (1.25)	32.33***
^a^Farmland proportion infested by FAW						4.74***
Mean	26.65	25.52	35.70	34.91	30.07	
Minimum	3	1	3	1	1	
Maximum	100	100	100	100	100	
Maize yield lost (kg ha^−1^)	487 (0.70)	459 (1.58)	610 (0.90)	626 (1.11)	536 (1.43)	6.34***

SD, standard deviation. ^a^Values are in percentages. ***1% significance level.

The proportion of farmland infested by FAW was significantly higher in the northern regions of the country (Guinea savannah and Sudan savannah) compared to the middle part (transitional and semi‐deciduous forest). This pattern suggests that the relatively drier conditions in the northern belt create a more favourable environment for the pest, supporting the findings from Ethiopia^30^ which reported that FAW thrives in drought and dry conditions. This highlights the importance of considering regional climatic factors when developing pest management strategies. All agroecological zones recorded farm infestations as high as 100%, with entire farmlands affected by the FAW reflecting the pest's invasive and devastating nature. A previous study^31^ highlighted that FAW can cause up to 100% yield loss in maize, further emphasising the significant threat it poses to the livelihoods and food security of many households.

The study further reports, as presented in Table 4, that yield loss due to FAW infestation varied significantly across the agroecological zones. The variation in yield losses across agroecological zones is consistent with an earlier study^17^ that also observed significant differences in output losses across agroecological zones in Kenya. Semi‐deciduous recorded the lowest yield loss (459 kg ha^−1^) whereas the highest yield loss was recorded at Sudan savannah (626 kg ha^−1^). On average, yield loss as a result of FAW infestation is estimated at 536 kg ha^−1^. Yield losses are a great threat to farmer livelihood and food security.^32^ This may imply that intensity of FAW infestation is agro‐geographically inclined, hence interventions should take into consideration geographical characteristics to be effective.

### 3.3 Perceptions of maize farmers on FAW infestation

Intrinsic factors, such as perceptions, play a vital role in the decision‐making process of farmers, influencing how they respond to risks/threats, outbreaks and coping strategies. A robust pest management approach considers both the perceptions of farmers and the strategies adopted.^6^ Therefore, understanding farmers' perceptions of FAW infestation is crucial in minimising effects.

Table 5 presents results on the perceptions (pooled sample) of respondents on FAW infestation. Perception statements were categorised into four thematic areas: FAW incidence, FAW threats/effects, FAW management and FAW control measures. Perception indices were estimated by obtaining weighted means from farmers' responses. Regarding FAW incidence, a mean perception score of 0.00 was recorded for the statement ‘presence of other hosts reduces FAW infestation,’ clearly indicating that most farmers are unaware of this concept. Recent studies have found that maize yields were higher despite FAW infestation when intercropped with beans, attributing this increase to the benefits of intercropping.^33^, ^34^ This highlights the need to raise awareness among farmers about effective management practices, such as intercropping, which can reduce the impact of FAW. While 53% of farmers believed that high rainfall reduces FAW populations, 36% disagreed and 11% were neutral. This majority perception aligns with a previous study^35^ which found a negative correlation between high rainfall and FAW infestation. This finding highlights the role of rainfall in reducing FAW infestations, offering valuable insights for developing climate‐based pest management strategies that can improve FAW control efforts. Regarding FAW threats and effects, maize farmers unanimously perceived FAW as a severe threat to production, with all respondents rejecting the idea that FAW is not a threat. This strong consensus, reflected in a high negative mean perception score of −0.98, aligns with earlier studies^7^, ^36^ that similarly reported that farmers associate FAW with significant economic losses in maize production, reinforcing the need for targeted pest management strategies to mitigate crop losses and safeguard food security. Similarly, there was a strong negative perception towards the idea that FAW cannot completely destroy crop fields or negatively impact maize yield and household food consumption, with mean scores of −0.93, −0.81 and −0.65, respectively. An overall perception index of −0.84 further underscores the strong negative sentiment regarding FAW threats and effects, aligning with findings from previous studies^37^, ^38^ that FAW is a highly destructive and devastating maize pest. The significant negative perception regarding household food consumption emphasises the critical role of FAW in exacerbating food insecurity, underlining the necessity for targeted interventions to protect both crop yields and household food supplies. Regarding FAW management, while 66% of respondents believed that early identification facilitates easier control, 25% disagreed, citing that once FAW is seen on the farm, they spread remarkably rapidly, hence early identification does not necessarily ease control. However, research supports that early identification helps minimise infestations by allowing prompt action, which can guide management strategies and reduce costs and time.^37^, ^39^, ^40^ This accentuates the importance and need for early detection strategies to effectively minimise crop damage. Further, the positive statements regarding cost, labour intensity and ease in management of the pest all recorded strong negative reactions, indicating that FAW is perceived to be cost‐effective, labour intensive and difficult to manage. This is consistent with earlier study,^12^ which noted that FAW control can cause direct capital costs through an increase in demand of skilled labour to manage the infestation. Earlier studies^30^ also highlighted that the pest does not only destroy crops but also comes with financial burdens because it is accompanied by loss of money for treatment hence capital intensive.

**Table 5:** Farmers' perception on FAW infestation (pooled)

Perception statement	Strongly agree (1)	Agree (0.5)	Neutral (0)	Disagree (−0.5)	Strongly disagree (−1)	Mean
**FAW incidence**						
Presence of other hosts reduce FAW infestation	50 (11)	42 (9)	276 (58)	71 (15)	35 (7)	0.00
High rainfall leads to reduced FAW infestation	139 (29)	114 (24)	53 (11)	78 (17)	90 (19)	0.14
***Perception index***						***0.07***
**FAW threats/effects**						
FAW is not a serious threat to maize production	–	–	–	24 (5)	445 (95)	−0.98
FAW can't destroy an entire crop field	–	3 (0.6)	2 (0.4)	56 (12)	408 (86)	−0.93
FAW can't affect your maize yield	11 (2)	21 (5)	7 (1)	56 (12)	379 (80)	−0.81
FAW infestation can't affect household food consumption	8 (2)	47 (10)	39 (8)	84 (18)	296 (62)	−0.65
***Perception index***						***−0.84***
**FAW management**						
FAW when identified early is easy to control	237 (50)	83 (18)	32 (7)	39 (8)	78 (17)	0.37
FAW management is not cost intensive	–	7 (1)	–	22 (5)	445 (94)	−0.96
FAW management is not labour intensive	–	3 (1)	7 (2)	53 (10)	411 (87)	−0.92
FAW infestation is easy to manage	7 (1)	–	–	22 (5)	445 (94)	−0.95
***Perception index***						***−0.62***
**FAW control measures**						
Relative to traditional and synthetic methods biological/botanical control is best	22 (5)	80 (17)	228 (50)	90 (19)	41 (9)	−0.05
Relative to biological/botanical and synthetic methods traditional control is best	49 (11)	124 (27)	185 (40)	71 (15)	34 (7)	0.09
Relative to traditional and biological methods synthetic control is best	201 (42)	116 (25)	115 (24)	32 (7)	10 (2)	0.49
Combination of the methods gives best results	123 (26)	115 (24)	167 (35)	49 (10)	20 (4)	0.29
***Perception index***						***0.21***

Values in parentheses represent percentage of total respondents.

The mean perception index for FAW control measures was 0.21, indicating a neutral stance among farmers, which suggests limited knowledge about effective control strategies. This finding aligns with earlier study,^41^ which reported similar knowledge gaps among maize farmers in Mozambique. The result underscores the need for more comprehensive training, specifically, programs that include repeated follow‐ups, participatory learning approaches and access to current pest information, ensuring that farmers not only acquire knowledge but are also able to apply it effectively under their specific farm conditions. Consistent with the widely observed overdependence of farmers on synthetic pesticides,^42^, ^43^, ^44^, ^45^ the majority of farmers (67%) believed that synthetic control methods offer better results relative to traditional or biological/botanical measures, citing synthetic applications to be less labour‐intensive and produce faster results. In Congo, farmers similarly perceive that without synthetic pesticides, it is impossible to grow crops without significant losses.^46^ However, earlier study^12^ opined that a judicious combination of management strategies yields better results than implementing lone measures. This finding accentuates the prevalent reliance on synthetic pesticides among farmers, underscoring the need for research into integrated FAW management strategies that combine effective and sustainable practices. Further, the importance of integrating diverse control measures, including synthetic, traditional and biological tactics, is emphasised for a holistic and sustainable approach to FAW management. It also emphasises the importance of educating farmers on balanced pest management approaches.

Understanding farmers' perceptions is crucial because it shapes decision‐making and informs the development of targeted solutions.^47^ The insights gained from this study on farmers' perceptions of FAW infestation are therefore valuable for designing effective interventions to mitigate the impact of this pest.

### 3.4 Factors influencing choice of FAW management strategy adopted

Table 6 presents the empirical model estimates from multivariate probit regression, capturing the factors influencing farmers' choice of management techniques. The chi‐square value indicates that the regression model estimated is significant at the 1% level, suggesting an appropriate fit to data. Additionally, the Wald test value reveals that the explanatory variables considered in the MVP model offer a convincing justification for the choice of management practices.

**Table 6:** Factors influencing the choice of management strategies adopted by farmers to control FAW

Factors	Management strategies					
	Synthetic measure		Biological measure		Traditional measure	
	d*y*/d*x*	Robust SE	d*y*/d*x*	Robust SE	d*y*/d*x*	Robust SE
Cons.		0.9459		0.4227		0.8180
Transitional zone	0.6636**	0.3393	1.5570**	0.7894	−1.2560**	0.5982
Semi deciduous zone	0.1524	0.5027	1.7285**	0.7037	−0.8274	0.5423
Guinea savannah	−1.1024**	0.4387	−0.9855***	0.4740	−0.1036	0.5040
Age	0.0063	0.0126	0.0787***	0.0274	0.0263**	0.0134
Sex	0.6638	0.3889	0.5697	0.7398	0.2999	0.4092
Household head status	0.9636***	0.3431	−0.4680	0.7236	−0.5177	0.3691
Household size	0.0299	0.0278	0.1199***	0.0450	−0.0590	0.0398
Marital status	0.1575	0.3570	−0.0906	0.5197	0.9721	0.5466
Years of education	0.0099	0.0273	0.1705***	0.0680	0.0595	0.0377
Farming experience	0.0282	0.0166	0.1250***	0.0361	0.0042	0.0153
Maize farm area	0.0692***	0.0275	−0.0800***	0.0266	−0.0024	0.0193
Land tenure	0.2301**	0.0998	0.2624	0.1709	0.3133***	0.0973
FBO membership	0.4842	0.3193	−0.7650	0.6809	−0.1361	0.3828
Extension access	0.1099	0.3086	−0.0767	0.3988	0.1081	0.3913
Training participation	0.5678**	0.2903	0.7790***	0.1270	−0.2456	0.3157
Credit access	0.0401	0.3208	0.6884	0.4344	−0.3156	0.3457
Off‐farm income	0.00009**	0.00004	0.00005	0.00007	0.00001	0.00007
Labour	−0.0026***	0.0009	0.0034**	0.0014	0.0009	0.0010

Wald *χ*2 = 1441.64 (df = 54, prob > *χ*2 = 0.0000). Likelihood ratio test of rho21 = rho31 = rho32 = 0: *χ*2 = 10.02 (df = 3, prob > *χ*2 = 0.0184). d*y*/d*x*, marginal effects; FBO, farmer‐based organization; SE, standard error. ***1% significance level; **5% significance level; *10% significance level.

At the 1% significance level, farmers in the transitional zone were more inclined to adopt synthetic and biological/botanical measures for controlling FAW, but less likely to use traditional methods. Additionally, farmers in the semi‐deciduous forest zone were 1.73 times more likely to choose biological/botanical control strategies compared to those in the Sudan savannah, which served as the reference location. Conversely, farmers in the Guinea savannah were less likely to adopt both synthetic and biological/botanical control methods, a difference that was also statistically significant at the 1% level. This implies that the adoption of FAW management practices is region‐specific, as evidenced by variations in control preferences across the different agroecological zones. Therefore, interventions should be tailored to the ecological characteristics of each region to be more effective. Older farmers were more likely to choose biological/botanical and traditional pest control methods than younger farmers, likely due to traditional methods being viewed as labour‐intensive and time‐consuming. This finding aligns with Kinuthia,^48^ who noted that younger farmers tend to prefer synthetic methods for their efficiency. The positive relationship between age and the use of botanical control could be attributed to older farmers' accumulated experience, making them more knowledgeable in the preparation and application of botanicals than younger farmers who may be less experienced, underscoring the need for age‐specific training and outreach programmes to ensure the effective dissemination of pest management strategies across different farmer demographics.

Farmers who were heads of households were more likely to use synthetic control measures because decision‐making around chemical use often rests with them in rural farming communities. Additionally, larger households were 0.1199 times more likely to adopt botanical methods for FAW control, likely due to the availability of additional labour for labour‐intensive tasks such as preparing and applying botanicals. Preparation of botanicals and their application is multi‐staged and considered labour intensive.^49^, ^50^ This finding underscores the influence of household structure on pest management decisions, highlighting the need for tailored interventions that consider household dynamics when promoting sustainable pest control strategies within rural farming systems. The study further reveals that with each additional year of formal education, farmers were 0.1705 times more likely to choose botanical control methods. Educated farmers are better equipped to research and implement biological FAW control solutions because they have improved access to information and the ability to process and apply that knowledge effectively. The positive influence of formal education on choice of botanicals may also be attributed to the fact that more educated farmers are better informed on dangers associated with inappropriate chemical use, leading them to favour safer and more environmentally friendly biological or botanical control methods. This aligns with previous studies^48^ which noted that formal education enhances a farmer's capacity to seek out safer alternatives, such as botanical methods, which are gaining recognition as environmentally friendly pest control solutions.^51^, ^52^ The finding emphasises the role of education in promoting sustainable FAW management practices among farmers.

An increase in the number of years a farmer has cultivated maize is strongly linked to a greater likelihood of using botanical measures to manage FAW infestation, with statistical significance at the 1% level. This suggests that more experienced farmers tend to prefer botanical control methods, likely due to their accumulated knowledge of diverse pest management strategies and a broader understanding of alternatives to synthetic chemicals. Guided interventions can thus promote sustainable pest management by leveraging the knowledge and experience of seasoned farmers to reduce reliance on synthetic chemicals. The result in Table 6 reveals that farmers with larger maize farms were less likely to adopt biological pest control strategies for managing FAW. This can be attributed to the fact that botanical pest control methods are more time‐consuming and labour‐intensive.^49^, ^50^ As farm size increases, the practicality of using biological methods decreases due to higher demands on time, cost and labour. Conversely, synthetic pest control becomes more feasible for larger farms because it is perceived to be faster, less time‐consuming and less labour‐intensive.^49^, ^53^ As larger farms tend to favour synthetic control methods due to the feasibility and efficiency required to manage larger plots, it is important to innovate more scalable and labour‐efficient biological control methods to enhance their adoption across farms of varying sizes. Farmers who own the land they cultivate are more likely to implement synthetic and traditional control strategies for managing FAW, with statistical significance at the 1% level. Landowners have the autonomy^53^ to test and apply chemicals or use traditional methods, such as burning and fallow periods, whereas those who rent or engage in sharecropping may face restrictions due to tenure limitations, emphasising the importance of considering land tenure status in developing FAW management strategies and policies, as it significantly influences the adoption of control strategies. The results also show that participation in pest and disease training significantly increases the likelihood of adopting both synthetic and biological control measures, with a much stronger effect on biological options (0.779) compared to synthetic pesticides (0.568). This indicates that training promotes more active FAW management overall, particularly the uptake of sustainable, non‐chemical practices. However, the simultaneous increase in synthetic pesticide use suggests that current training may still reinforce farmers' reliance on chemical control. These patterns point to gaps in training content and delivery, highlighting the need to reorient training toward practical IPM approaches emphasising safe pesticide use and the effective application of biological, botanical and traditional methods. Interestingly, while training strongly shapes pest management decisions, extension contact shows no significant effect on the adoption of any management strategies, suggesting that extension interactions as currently delivered may not be sufficiently impactful on farmers' management decisions. The significant role of training, despite the non‐significant influence of extension contact, underscores the important but underutilised role of extension agents. This highlights the need to reorient extension efforts and strengthen the capacity of extension agents through up‐to‐date knowledge on FAW management and participatory training approaches, to better guide farmers toward environmentally friendly and economically viable practices. Further, farmers with higher non‐farm incomes were more likely to use synthetic control measures for managing FAW infestations. This is likely because synthetic insecticides are relatively costly^55^, ^56^ and higher non‐farm income enhances affordability. Additionally, increased non‐farm income may lead to more time spent away from the farm, prompting farmers to choose less time‐consuming options, such as synthetic pesticides, despite their higher cost. This finding is consistent with a previous study^57^ that noted that off‐farm income is positively associated with increased pesticide use. Farmers' income and time constraints affect their pest management choices, so interventions should be tailored to accommodate varying income levels and time availability to promote sustainable FAW infestation solutions. Labour availability had a significant impact on pest control choices, with limited labour leading farmers to prefer synthetic methods, which are less labour‐intensive and yield faster results.^54^ This aligns with an earlier study^49^ that found that farmers view chemical insecticides as convenient, requiring minimal labour. In contrast, farmers with greater labour access were more inclined to use botanical control methods, likely due to the labour‐intensive nature of preparing and applying botanicals.^49^, ^50^ This underscores the need to develop and promote FAW control measures that are both effective and labour‐efficient, particularly for farmers with limited labour resources.

### 3.5 Constraints faced by maize farmers in managing FAW infestation

Table 7 shows the ranking of challenges faced by farmers in controlling FAW infestation across the agroecological zones. The significant estimated chi‐square (*χ2*) indicates that the order of ranking of constraints is robust. Also, the Kendall's coefficient of concordance (*W* ^*b*^) value of 0.541 implies that with regards to the pooled sample, there is a 54.1% agreement in the ranking of the constraints among farmers. The rankings indicate that farmers’ most pressing challenge was difficulty accessing synthetic measures, with a mean rank of 1.88. The second major constraint was the high cost of FAW control, particularly linked to purchasing synthetic chemicals according to farmers. Inadequate knowledge in managing FAW infestations ranked third, as many farmers admitted to using pesticides based on hearsay rather than informed decisions. The fourth most pressing issue was limited access to biological/botanical measures, reinforcing the need for training focused on safer, more effective pest control alternatives. The least pressing constraint among farmers was limited access to extension services. Interestingly, this aligns with our regression results, which show that extension access was not a significant determinant of farmers’ FAW management choices. It appears extension officers have lost credibility among farmers in helping them solve their FAW challenges. This is concerning because effective extension services are crucial for promoting agricultural production and technology adoption. The situation is exacerbated by the high extension‐to‐farmer ratio in Ghana, estimated at 1 extension agent per nearly 1900 farmers,^29^ which severely impacts the efficiency and effectiveness of these services. To improve pest management and overall agricultural productivity, the government needs to enhance extension services by increasing manpower, providing training and equipping extension agents with necessary tools to strengthen the relevance and quality of extension support.

**Table 7:** Ranking of constraints faced by farmers in controlling FAW across agroecological zones

Constraints	Agroecological zones				
	Transition (*n* = 151)	Semi‐deciduous forest (*n* = 135)	Guinea savannah (*n* = 93)	Sudan savannah (*n* = 95)	Pooled (*N* = 474)
	Rank (mean)	Rank (mean)	Rank (mean)	Rank (mean)	Rank (mean)
High cost associated with FAW control	***2nd*** (3.98)	***2nd*** (3.58)	***2nd*** (3.33)	***2nd*** (2.81)	***2nd*** (3.48)
Lack of technical know‐how in identifying larvae	***6th*** (4.59)	***6th*** (4.86)	***6th*** (4.90)	***5th*** (4.68)	***6th*** (4.75)
Inadequate knowledge in managing the infestation	***3rd*** (4.01)	***3rd*** (3.97)	***4th*** (4.20)	***3rd*** (4.39)	***3rd*** (4.12)
Difficulty in accessing biological/botanical measures	***4th*** (4.09)	***4th*** (4.29)	***3rd*** (4.07)	***6th*** (4.80)	***4th*** (4.30)
Difficulty in accessing traditional methods	***5th*** (4.41)	***5th*** (4.38)	***5th*** (4.56)	***7th*** (4.97)	***5th*** (4.56)
Difficulty in accessing synthetic measures	***1st*** (2.08)	***1st*** (1.72)	***1st*** (1.66)	***1st*** (2.00)	***1st*** (1.88)
Limited access to extension advice	***7th*** (4.83)	***7th*** (5.19)	***7th*** (5.29)	***4th*** (4.35)	***7th*** (4.91)
Kendall's *W* ^*b*^	*0.280*	*0.583*	*0.619*	*0.583*	*0.541*
*χ*2	*130.44*	*201.91*	*149.23*	*158.02*	*593.50*

df = 6; asymp. sig. = 0.000.

In conclusion, the primary challenges in FAW control revolve around accessibility, affordability, availability and inadequate knowledge. The identified challenges related to the affordability and accessibility of synthetic control measures for FAW underscore the need for a targeted recommendation. This presents an opportunity to develop and promote cost‐effective, environmentally friendly alternatives that are both effective and accessible, helping dispel the perception that synthetic pesticides are inherently superior and reduce overreliance on them. This approach should involve a multifaceted effort to subsidise costs while also investing in the development and promotion of sustainable, bio‐based alternatives. Ensuring that these sustainable measures are readily available and economically accessible to farmers will require collaboration between government bodies, researchers, agricultural organisations and the private sector. This will enable farmers to adopt effective FAW management practices without facing financial barriers, ultimately contributing to more sustainable agricultural practices.

## 4 CONCLUSIONS

Overall, this study provides an in‐depth examination of Ghanaian farmers' perceptions of FAW infestations, their management strategies and the factors influencing the adoption of these strategies. It reveals that pest control practices vary significantly across agroecological zones, suggesting the need for region‐specific strategies. Farmer perceptions reveal a predominant reliance on synthetic chemical controls over botanical or traditional methods, driven by generally limited awareness and knowledge of FAW management practices. The study also reveals a divergence in farmer perceptions about the effectiveness of early FAW identification. While many farmers believe early detection simplifies control efforts, a significant portion also argue that the rapid spread of FAW diminishes the benefits of early identification, suggesting a gap between perceived and actual efficacy. More experienced and older farmers are inclined towards botanical and traditional control methods, reflecting their accumulated knowledge and preference for diverse pest management approaches. Conversely, higher non‐farm income increases the likelihood of using synthetic pesticides, while limited labour availability drives the preference for less labour‐intensive options. Moreover, the study finds that higher levels of formal education increase the likelihood of farmers opting for botanical control measures because educated farmers are better informed about the benefits and safer use of biological options. Additionally, land ownership influences pest control choices, with landowners more likely to adopt both synthetic and traditional methods compared to renters or sharecroppers. The study also highlights that labour availability affects control choices, with limited labour pushing farmers towards synthetic methods and more labour encouraging biological/botanical measures. Major constraints identified include high costs, limited access to effective control measures and inadequate knowledge of FAW management strategies.

However, the study acknowledges limitations due to its reliance on cross‐sectional data, which may not fully capture the dynamics of FAW infestation across different agricultural seasons. It suggests that future research adopt a longitudinal approach for a more detailed understanding of FAW management over time.

## Data Availability

The data that support the findings of this study are available on request from the corresponding author. The data are not publicly available due to privacy or ethical restrictions.
